# The value of ultrasound and magnetic resonance imaging scoring systems in explaining handgrip strength and functional impairment in rheumatoid arthritis patients: a pilot study

**DOI:** 10.1007/s11547-022-01499-0

**Published:** 2022-05-14

**Authors:** Fausto Salaffi, Marina Carotti, Marco Di Carlo, Luca Ceccarelli, Sonia Farah, Andrea Giovagnoni

**Affiliations:** 1 Rheumatology Clinic, Dipartimento Di Scienze Cliniche e Molecolari, Università Politecnica delle Marche, Ospedale “Carlo Urbani”, Jesi (Ancona), Italy; 2 Dipartimento di Scienze Radiologiche, Ospedali Riuniti, Università Politecnica delle Marche, Ancona, Italy; 3 Department of Interventional and Diagnostic Radiology, Azienda Ospedaliero-Universitaria Sant’Anna, Ferrara, Italy

**Keywords:** Rheumatoid arthritis, Handgrip strength, Disease activity, Magnetic resonance imaging, Ultrasound

## Abstract

**Purpose:**

The goal of this study is to investigate the relationship between joint inflammation and damage of the wrists and hands, measured by semiquantitative ultrasound and magnetic resonance imaging scoring systems, with functional disability and handgrip strength (HGs).

**Materials and methods:**

Consecutive adult RA patients with active disease, as defined by a Disease Activity Score 28 joints C-reactive protein (DAS28-CRP) > 3.2, underwent a cross-sectional evaluation comprehensive of a clinimetric assessment, an HGs evaluation, an ultrasound assessment aimed at calculating the UltraSound-CLinical ARthritis Activity (US-CLARA), and a magnetic resonance imaging scored according to the modified Simplified Rheumatoid Arthritis Magnetic Resonance Imaging Score (mod SAMIS). The Spearman’s rho correlation coefficient was used to test the correlations.

**Results:**

Sixty-six patients with RA were investigated (age 55.6 ± 12.2 years). The mod SAMIS total score and the US-CLARA had a weak but significant correlation (rho = 0.377, *p* = 0.0018). Among the mod SAMIS sub-scores, there was a significant relationship between mod SAMIS bone edema (SAMIS-BME) and US-CLARA (rho = 0.799, *p* < 0.001) and mod SAMIS synovitis (SAMIS synovitis) and US-CLARA (rho = 0.539, *p* < 0.001). There were also significant negative relationships between the HGs score and the mod SAMIS total score and US-CLARA (rho = − 0.309, *p* = 0.011 and rho = − 0.775, *p* < 0.0001, respectively).

**Conclusions:**

BME and synovitis have an influence on the function of the upper extremities. The US-CLARA and the mod SAMIS total score are intriguing options for semiquantitative assessment of joint inflammation and damage in RA.

## Introduction

Rheumatoid arthritis (RA) is a chronic inflammatory joint disease that mostly affects the hands, resulting in pain, deformity, and functional limitations. Inflammation of small joints can lead to articular abnormalities and muscle weakness, with consequent impairment of activities of daily living (ADLs) [1, 2]. It is estimated that 70% of RA patients show some hand dysfunction during their disease [3]. An adequate handgrip strength (HGs) is fundamental for the majority of ADLs [4]. A number of studies have shown that chronic, low-grade inflammation has a role in the deterioration of HGs [5]. On the other hand, the traditional disease activity scores such as the Disease Activity Score with 28-joint count (DAS28) may not accurately reflect the regional impact of RA on the hands. The majority of research in this topic has focused on individuals with advanced RA who were not treated according to contemporary guidelines in the early stages of the disease [6, 7].

Nowadays, imaging modalities such as ultrasound (US) and magnetic resonance imaging (MRI) play a significant role in the care of RA patients, in addition to clinical assessment. The great sensitivity of US to detect joint abnormalities is recognized in current European League Against Rheumatism (EULAR) recommendations on the use of imaging modalities in RA [8], advising that this technique should be used for an accurate assessment of patient’s disease activity [9]. In particular, the sensitivity of power Doppler ultrasound (PDUS) in detecting inflammatory flow at the microvascular level has proven to be a useful technique for quantifying the degree of inflammation in RA [10, 11]. For the detection of early bone damage, MRI has shown to be more sensitive than traditional radiography [12]. Furthermore, MRI has the ability to visualize synovitis and bone marrow edema (BME), which has been demonstrated to be predictive of radiologic development [13–15]. Treatment benefits on joint inflammation have been observed using periodic MRI examination in studies lasting 3–6 months [13, 16, 17]. The Food and Drug Administration and the European Medicines Agency guidance documents admit that MRI measures may be useful for assessing RA joint damage in randomized controlled trials (RCTs), but they also state that MRI methodologies are not properly validated [18, 19].

The standardized scoring systems for quantifying inflammatory signs and/or joint damage in RA using different imaging techniques are rapidly evolving, with the development of the Rheumatoid Arthritis Magnetic Resonance Imaging Score (RAMRIS) [20] and Simplified Rheumatoid Arthritis Magnetic Resonance Imaging Score (SAMIS) systems for MRI [21], and the EULAR-Outcome Measures in Rheumatology Clinical Trials (OMERACT) system for US [22, 23]. According to the OMERACT filter, RAMRIS and SAMIS synovitis, osteitis, and erosions seen with 1.5 T MRI, are valid and useful for assessing joint inflammation and damage in RA of the wrist/hand [24, 25].

US scoring systems have been also implemented in multimodal disease activity indices. The construct validity and reliability of the UltraSound-CLinical ARthritis Activity (US-CLARA) index, which combines the values of the Recent-Onset Arthritis Disability (ROAD) questionnaire, self-administered tender joint count (TJC) scores, and the US assessment into a single measure of disease activity for RA have been investigated. US-CLARA demonstrated a strong correlation with the traditional disease activity indices [DAS28, Simplified Disease Activity Index (SDAI), and Clinical Disease Activity Index (CDAI)] [26, 27].

Starting from these considerations, the goal of this research is to investigate the relationship between joint inflammation and damage of the wrists and hands, measured by semiquantitative US and MRI scoring systems, with functional disability and HGs.

## Methods

### Design and study population

This pilot study included consecutive adult RA patients, defined according to the 2010 American of College of Rheumatology (ACR)/EULAR criteria [28], with an active disease, as defined by DAS28 C-reactive protein (DAS28-CRP) > 3.2, independently of current therapy. Exclusion criteria were represented by the coexistence of comorbid conditions able to interfere with the clinical and HGs assessment: coexisting fibromyalgia, hearth failure, severe chronic obstructive pulmonary disease, multiple sclerosis, Alzheimer disease, extracorporeal dialysis, active neoplasms, or persistent infectious diseases.

### Demographics, clinical and composite disease activity assessment

Data on demographic characteristics and all core-set variables were extrapolated from the internal center database. These details included age, gender, and the length of the illness (defined as time since diagnosis). For each patient was collected the presence of rheumatoid factor (RF), anti-citrullinated protein antibodies (ACPA), and CRP.

The following items of disease activity indices were used in clinical assessments: 28-joint counts for swollen and tender joints (SJC and TJC, respectively), patient self-administered tender joint count (self-TJC), numerical rating scale (NRS) of pain, evaluator, and patient assessments of disease activity (EGA and PGA, respectively) by NRS, patient assessment of general health status by NRS (GH). Composite disease activity indices, such as the DAS28, the CDAI, and the SDAI, were calculated using these variables [29–32].

### Assessment of physical functioning with patient-reported outcome measures (PROs)

All patients completed the shortened Disability of Arm, Shoulder and Hand questionnaire (QuickDASH) [33], the hand and finger function subscale of the Arthritis Impact Measurement Scale (AIMS2-HFF) [34, 35], and the upper extremity function of the ROAD questionnaire [36, 37].

#### The shortened Disability of Arm, Shoulder and Hand questionnaire (QuickDASH)

The QuickDASH is a patient-based outcome instrument for assessing upper extremity function [33]. It is a shortened version of the original DASH outcome measure [38], which assesses a person's capacity to execute tasks, absorb pressures, and the severity of their symptoms [39]. Compared to the original DASH outcome measure, which included 30 elements, the QuickDASH only has 11 items. The QuickDASH tool is made of 5-point Likert scales, with the patient selecting a number that corresponds to his or her severity/function level [40–42]. The given values for all completed replies are simply added together and averaged to get a five-point score. After removing one and multiplying by 25, this result is converted to a score out of 100. This adjustment is used to make the score more comparable to other 0–100 scaled measurements. A higher score implies a higher level of impairment. For this study was employed the Italian QuickDASH validated version [43].

#### The Hand and Finger Function subscale of the Arthritis Impact Measurement Scale (AIMS2-HFF)

The AIMS2-HFF was created with the goal of evaluating physical function in individuals with rheumatic diseases. The AIMS2 is an updated and expanded version of the AIMS, is a self-administered questionnaire that assesses three areas of one’s health: physical, psychological, and social [34]. For the purposes of this study, it has been used only the physical domain questions, namely ones concerning hand and finger function. On a 5-point Likert scale, the patients were asked how often they experienced impaired hand and finger function when completing five particular tasks: writing with a pen or pencil; buttoning up a shirt; turning a key; tying knots or shoelaces; and opening a jar within the preceding four weeks. Each of the item’s scores, which ranged from 1 (every day) to 5 (never), were combined to create a total score, which ranged from 0 (indicating excellent function) to 10 points (representing poor function). The Italian validated version of AIMS2 demonstrated good metrologic properties [35, 44].

#### The Recent-Onset Arthritis Disability (ROAD) questionnaire

The ROAD is a valid, reliable, and responsive instrument for assessing physical function in RA patients [36, 37]. The ROAD is a 12-item questionnaire and contains questions about fine upper extremity movements, lower extremity activities, and tasks that include both upper and lower extremities. Patients are asked to rate the amount of difficulty during the previous week on a 5-point scale ranging from 0 (no difficulty) to 4 (extreme difficulty, unable to do). The ROAD score has a range of 0 to 48, with a simple mathematical normalizing process the score is converted to a 0–10 scale (higher scores indicating worse physical function). A previous study using both classical test theory and Rasch analysis methods supported the use of separate sub-scores for upper limb function, lower limb function, and activities of daily living/work [45]. In this study, only the ROAD upper extremity function sub-score was calculated.

### Assessment of handgrip strength (HGs)

**Fig. 1:**
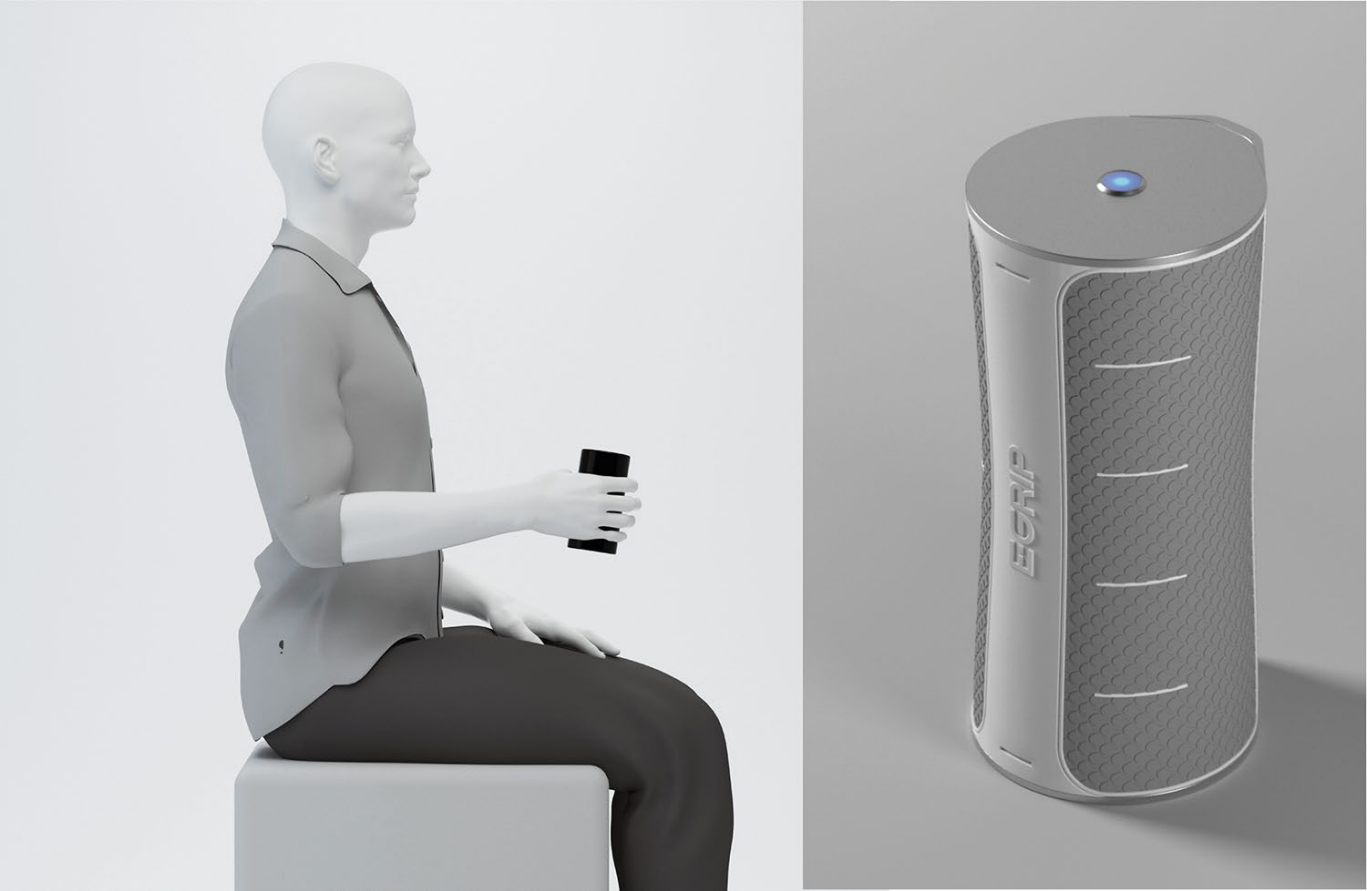
Assessment of handgrip strength (HGs). In the seated position, HGs was measured with the shoulder adducted and neutrally rotated. The wrist was slightly extended, the elbow was flexed to roughly 90 degrees, and the forearm was in neutral. Instructions were presented in a consistent manner

HGs was assessed using a cylindrical-shape grip device with five force sensors (FSR-402, interlink electronics and connected to an Arduino Mega 2560). This instrument records peak force data as a continuous acquisition within a frame of 30 s with one measurement per sensor every 5 s, providing information on maintained grip rather than a single-time peak of grip force [26, 46]. HGs was measured twice in the dominant hand, with the average of the two results utilized, and with a 5 min interval between the two measurements to recover from muscle weariness [47]. For subject placement, the American society of hand therapist’s instructions were followed [48] (Fig. 1).

### Joint inflammation and damage assessment

Semiquantitative assessments of joint inflammation and damage were carried out using US and MRI scoring techniques such as US-CLARA [27] and mod SAMIS [21].

#### The UltraSound-CLinical ARthritis Activity index (US-CLARA)

**Fig. 2:**
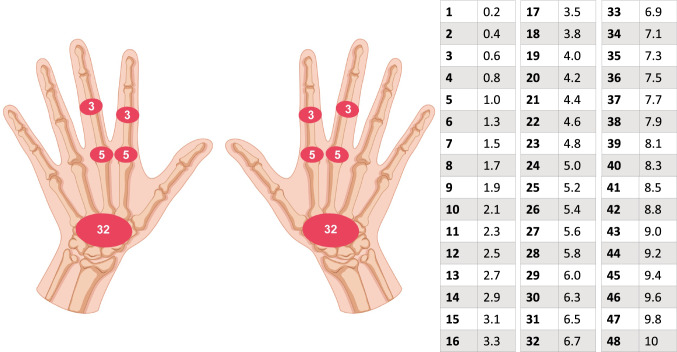
The UltraSound-CLinical ARthritis Activity (US-CLARA) scoring spreadsheet. Weight of each joint according to the Thompson's articular index and nomogram. The US final score is the sum of the weights of the joints of both hands divided by two (range 0–48), the value is normalized from a 0–48 scale to a 0–10 scale, using the nomogram

The US-CLARA is a composite index that combines the ROAD [36, 37, 45], a self-administered TJC, and the US semiquantitative evaluation into a single disease activity measure. Its total score ranges from 0 to 10 and was calculated by adding the scores of the three distinct metrics and dividing by three [27]. The self-administered TJC is that of RA Disease Activity Index (RADAI) [49]. The US examination includes multiplanar gray scale (GS) and power Doppler (PD) dorsal scans of both wrists and hands, examining the following joints: radiocarpal, 2nd and 3rd metacarpophalangeal (MCP), and 2nd and 3rd proximal interphalangeal (PIP) (Fig. 2). The results of the US exams are weighted by joint area according to Thompson’s articular index [50] and then normalized on a scale of 0–10. For US-CLARA, the following interpretability cutoff values have been proposed: remission (REM) US-CLARA < 2.0; low disease activity (LDA) 2.0 ≤ US-CLARA < 3; moderate disease activity (MDA) 3 ≤ US-CLARA ≤ 4.8; high disease activity (HDA) US-CLARA > 4.8. The detailed description of the index is provided in the original paper [27].

#### MRI scanning and modified SAMIS simplified score (mod SAMIS)

**Fig. 3:**
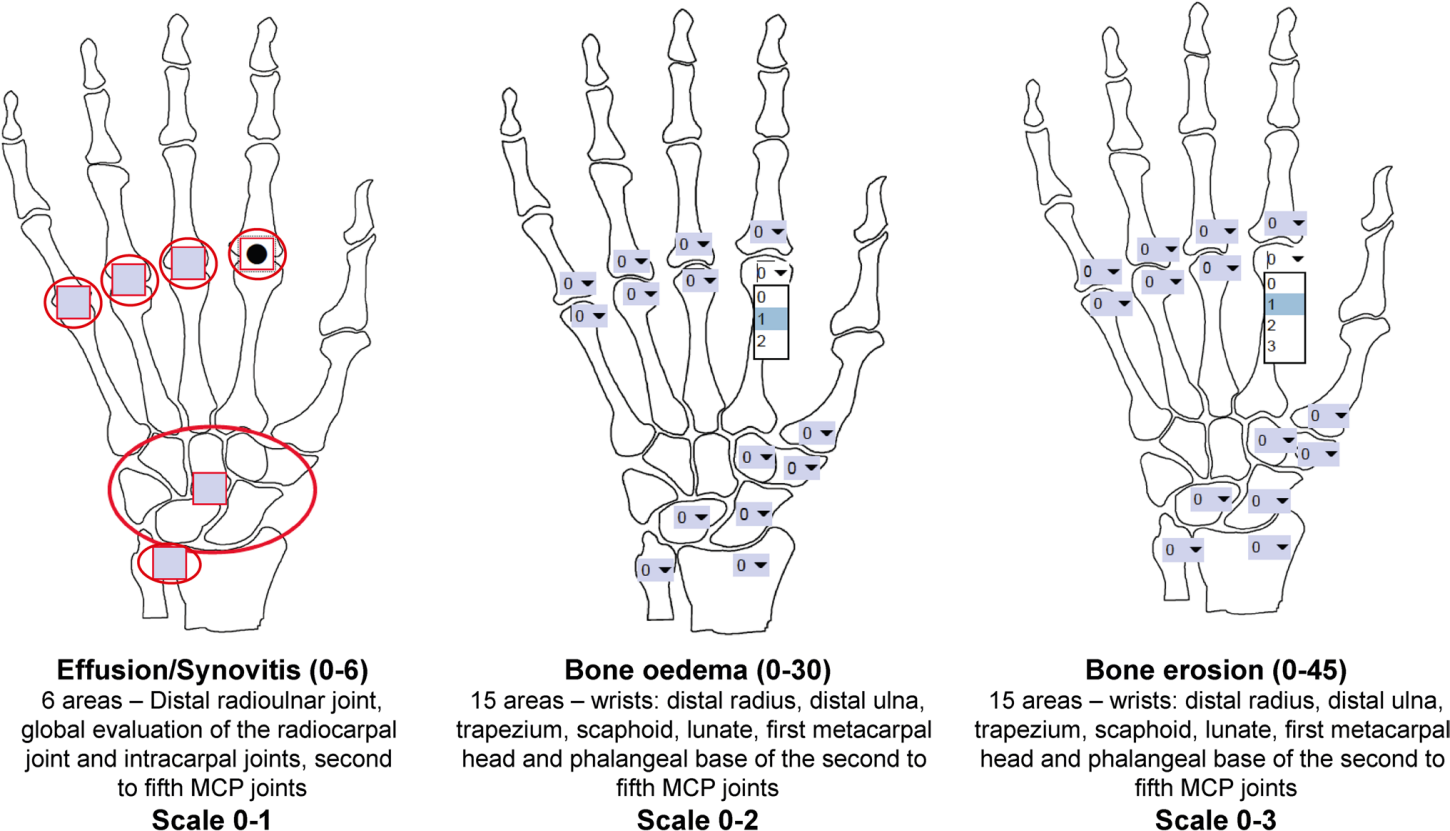
The modified simplified SAMIS magnetic resonance score (mod SAMIS) scoring spreadsheet. The MRI was graded for the presence/absence of synovitis and semiquantitative ratings of bone marrow edema and bone erosion, without contrast injection

The MRI was performed using a 1.5 T Impact MRI device (Achieva Philips Medical Systems, Best, The Netherlands) with four phased-arrived coils that were exclusively received. The sequences were taken without the use of contrast and are detailed in Table 1. The SAMIS was realized to reduce MRI scoring time while maintaining correlation with the OMERACT RA-MRI scoring system and similar or superior intra- and inter-reader reliability [21, 51]. SAMIS evaluates only one hand and was based on the radiographic Simple Erosion Narrowing Score (SENS) [52], reducing the number of study areas from 116 to 36. Erosions were scored with a scale from 1 to 10. Edema and synovitis were scored with scales from 0 to 2 and 0 to 1, respectively. The scoring system can be distinct in three sub-scores evaluating the presence/absence of synovitis (SAMIS synovitis), semiquantitative ratings for bone erosion (SAMIS-ERO), and bone marrow edema (SAMIS-BME), without contrast injection (Fig. 3).

**Table 1:** MRI sequence details

Sequence plane	Parameters
STIR coronal	FOV = 160 × 140, Matrix = 232 × 147, NEX = 3, slice thickness = 3 mm, gap = 0,3 mm, TR = 2500 ms, TE = 60 ms, TI = 140 ms
T1 TSE coronal	FOV = 260 × 140, Matrix = 520 × 220, NEX = 3, slice thickness = 3 mm, gap = 0,3 mm, TR = 566 ms, TE = 10 ms, flip angle = 90°
T1 TSE sagittal	FOV = 260 × 110, Matrix = 520 × 170, NEX = 3, slice thickness = 3 mm, gap = 0,3 mm, TR = 566 ms, TE = 10 ms, flip angle = 90°
STIR axial	FOV = 100 × 138, Matrix = 124 × 132, NEX = 2, slice thickness = 4 mm, gap = 0,4 mm, TR = 3684 ms, TE = 60 ms, TI = 140 ms
T2 TSE axial	FOV = 100 × 140, Matrix = 200 × 190, NEX = 3, slice thickness = 4 mm, gap = 0,4 mm, TR = 2760 ms, TE = 90 ms, flip angle = 90°
T2 GRE coronal	FOV = 260 × 160, Matrix = 520 × 257, NEX = 1, slice thickness = 3 mm, gap = 0,3 mm, TR = 450 ms, TE = 12 ms, flip angle = 90°

*FOV* field of view; *NEX* number of excitations; *SE* spin echo, *TR* repetition time; *TE* echo time; *TI* inversion time; *T1 TSE* T1-weighted turbo spin echo; *STIR* short tau inversion recovery; *T2 GRE* T2-weighted gradient echo

Only one hand was assessed by MRI. If both hands were equally painful it was evaluated the dominant one. According to van der Heijde radiographic scoring system, several bones were ruled out for ERO and BME [53]. The metacarpal head and phalangeal base from the 2nd to the 5th MCP joints, as well as the first metacarpal base, trapezium, scaphoid, lunate, and distal end of both the ulna and radius, were also investigated. ERO was scored in proportion of eroded bone to the measured bone volume on a 0–3 scale: (0) no erosion; (1) 11 to 33% of bone eroded; (2) 33 to 66% of bone eroded; (3) more than 66% of bone eroded. A BME scale ranging from 0 to 2 was applied to rate the fraction of bone that was edematous: 0 for normal bone, 1 for mild BME, and 2 for severe BME. Synovitis manifests itself as a region in the synovial compartment with an elevated signal on T2-weighted fat-suppressed images and a thickness higher than the typical synovium’s breadth. The distal radioulnar joint, the radiocarpal and intracarpal joints, and the 2nd to 5th MCP joints were all evaluated for the presence or absence of synovitis without being rated.

To determine interobserver variability, two radiologists (L.C., M.C.), who were blinded to clinical information and the other reader’s scoring, independently evaluated 22 cases within the same period by using MR imaging definitions of synovitis, BME and ERO, in accordance with the OMERACT RAMRIS recommendations [20].

### Statistical analysis

The data was input into a Microsoft Excel database and analyzed with MedCalc® 64-bit version 19.0.1.0. (MedCalc Software, Mariakerke, Belgium). The sample size for a pilot trial like this one should be at least 40 patients, according to standards (54). Where available, median and interquartile ranges, as well as means and standard deviations, are displayed (SD). Considering that data were not normally distributed (Kolmogorov–Smirnov test for normal distribution), nonparametric procedures have been used in order to provide a more cautious estimate of statistical significance.

Data deriving from US and MRI have been compared with the other clinimetric measurements employed in the study (DAS28-CRP, SDAI, CDAI, ROAD upper extremity function, AIMS2-HFF, QuickDASH, HGs). To quantify the correlations, it has been used the Spearman’s rho correlation coefficient, interpreted as follows: below 0.19 very weak; 0.20–0.39 weak; 0.40–0.59 moderate; 0.60–0.79 strong, above 0.79 very strong.

Intraclass correlation coefficient (ICC) average values with 95% confidence intervals (CIs) were used to report interobserver agreement. The ICC was considered as excellent if above 0.75, as fair to good if between 0.4 and 0.75, and as poor if below 0.4 [54].

## Results

### Demographic and pharmacological data

The study included 66 RA patients. The case study was composed mostly by middle-aged females [58 (87.9%), mean ± SD age 55.5 ± 12.2 years], with 10.6 ± 3.6 years of formal education, a mean disease duration of 4.4 ± 3.0 years. Fifty-seven (86.4%) patients were RF positive and 54 (81.8%) ACPA positive. The mean number of comorbidities was 1.7 comorbidities. All of the patients received at least one conventional disease-modifying anti-rheumatic drug (csDMARD) (methotrexate, leflunomide, sulfasalazine, or hydroxychloroquine) and/or a biological DMARD [39 (59.1%) patients, respectively, 15 (38.5%) adalimumab, 12 (30.8%) etanercept, 5 (12.8%) abatacept, 4 (10.2%) golimumab, and 3 (7.7%) tocilizumab]. Twenty-nine (43.9%) patients were taking oral corticosteroids, with a mean prednisone or equivalent dosage of 3.9 mg/day (range 2.5–25).

### Clinimetric and instrumental evaluation

The mean values (± SD) of DAS28, CDAI, and SDAI were, respectively, 4.54 ± 0.56, 35.92 ± 16.13, and 43.14 ± 14.91. The mean values of HGs peak grip force, ROAD upper extremity function, and QuickDASH, respectively, are 20.35 ± 9.17, 5.35 ± 2.80, and 26.66 ± 11.88. The SAMIS-ERO mean score was 18.28 ± 9.40_._ For the SAMIS-BME the mean score was 7.77 ± 4.90. The lunate, the capitate, the triquetrum, the hamate, the distal ulna, and the radius had the greatest SAMIS-ERO score, whereas the 2nd phalangeal base had the highest SAMIS-BME score, followed by the lunate, the capitate, the 4th metacarpal base, and the triquetrum. The SAMIS-synovitis mean score was 3.55 ± 1.87. The following bones were studied: distal radioulnar joint, global evaluation of the radiocarpal joint and intracarpal joints, second to fifth MCP joints. Mod SAMIS total score and US-CLARA mean values were determined to be 29.61 ± 11.29 and 5.76 ± 2.02, respectively. Table 2 lists the baseline characteristics of the 66 RA patients who took part in the trial.

**Table 2:** Demographic, clinical, and instrumental characteristics of 66 RA patients

	Mean	Median	SD	Q25–Q75
Age (years)	55.56	57.50	12.21	47.00–65.00
Educational level (years)	10.59	10.00	3.64	6.50–13.00
Disease duration (years)	4.43	4.00	3.00	3.00–7.00
Number of comorbidity	1.71	1.00	1.51	1.00–2.00
CRP (mg/dl)	4.47	3.41	3.56	2.20–4.84
TJC (0–28)	13.43	11.00	8.21	6.00–20.00
SJC (0–28)	9.59	8.00	6.45	4.00–14.00
GH (0–100)	70.07	80.00	27.25	50.00–90.00
DAS28-CRP (0–10)	4.54	4.60	0.56	4.09–4.99
CDAI (0–68)	35.92	33.00	16.13	23.00–49.00
SDAI (0–78)	43.14	42.05	14.92	30.55–55.66
HGs peak grip force (kg)	20.35	16.77	9.17	11.82–30.27
ROAD upper extremity function (0–10)	5.35	6.00	2.80	3.00–7.50
QuickDASH (0–100)	26.66	24.00	11.89	14.00–37.00
AIMS2-HFF	4.02	4.00	1.90	2.50–5.00
mod SAMIS-BME (0–30)	7.77	7.91	4.90	3.00–12.00
mod SAMIS-ERO (0–45)	18.28	18.61	9.40	12.00–22.00
mod SAMIS synovitis (0–6)	3.55	3.29	1.87	2.00–5.00
mod SAMIS total score (0–81)	29.61	30.00	11.29	21.50–36.00
US-CLARA (0–10)	5.76	6.53	2.02	3.76–7.30

*CRP* C-reactive protein; *TJC* tender joint count; *SJC* swollen joint count; *GH* general health status; *DAS28* Disease Activity Score 28 joints; *CDAI* Clinical Disease Activity Index; *SDAI* Simplified Disease Activity Index; *HGs* handgrip strength; *ROAD* Recent-Onset Arthritis Disability questionnaire; *QuickDASH* shortened Disability of Arm, Shoulder and Hand questionnaire; *AIMS2-HFF* Arthritis Impact Measurement Scale Hand and Finger Function; *mod SAMIS* modified Simplified Rheumatoid Arthritis Magnetic Resonance Imaging Score; *BME* bone marrow edema; *ERO* erosion; *US-CLARA* UltraSound-CLinical ARthritis Activity index

### Correlations and interobserver agreement

**Fig. 4:**
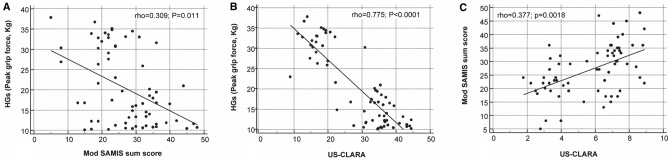
Scatterplot with linear regression lines displays the relationship between **A** mod SAMIS total score versus HGs, **B** US-CLARA versus HGs, and **C** US-CLARA versus mod SAMIS total score. The values of one variable appear on the horizontal axis, and the values of the other variable appear on the vertical axis. Each individual in the data appears as a point on the graph

US-CLARA was strongly and negatively correlated to the HGs, more than the mod SAMIS total score (rho 0.775; *p* < 0.0001 vs. rho 0.309; *p* = 0.011) (Fig. 4a, b). The mean HGs were very strongly and inversely correlated with the mod SAMIS-BME (rho 0.815; *p* < 0.0001), as well as with the mod SAMIS-synovitis score (rho 0.815; *p* < 0.0001).

A weak correlation was observed between mod SAMIS total score and US-CLARA (rho 0.377; *p* = 0.0018) (Fig. 4c).

The SAMIS-BME and SAMIS-synovitis scores were also linked with hand-specific self-report questionnaires (*p* < 0.05) as well as composite disease activity indicators such DAS28-CRP, CDAI, and SDAI (*p* < 0.0001). SAMIS-ERO had no relationship with HGs, functional impairment as determined by hand-specific self-report questionnaires, or composite disease activity indices. The mean HGs had a significant relationship with the values of hand-specific self-report questionnaires (*p* < 0.0001), as well as composite disease activity indices (*p* < 0.0001). Table 3 summarizes all the correlations studied.

**Table 3:** Spearman’s correlation coefficients among mod SAMIS scores, US-CLARA, HGs, disease composite activity indices, and hand-specific self-report questionnaires in patients with RA

	mod SAMIS-BME	mod SAMIS synovitis	mod SAMIS total score	US-CLARA	HGs peak grip force (kg)	CDAI	DAS28-CRP	SDAI	AIMS2 hand finger function	Quick DASH	ROAD upper extremity function
mod SAMIS-ERO	0.042 0.7335*	− 0.174 0.1560*	0.798 < 0.0001*	0.054 0.6680*	0.107 0.3925*	− 0.010 0.9393*	− 0.030 0.8087*	− 0.135 0.2794*	− 0.079 0.5290*	− 0.119 0.3393*	− 0.094 0.4517*
mod SAMIS-BME		0.626 < 0.0001*	0.477 < 0.0001*	0.799 < 0.0001*	− 0.815 < 0.0001*	0.678 < 0.0001*	0.715 < 0.0001*	0.588 < 0.0001*	0.345 0.0046*	0.312 0.0109*	0.809 < 0.0001*
mod SAMIS synovitis			0.288 0.0172*	0.539 < 0.0001*	− 0.711 < 0.0001*	0.489 < 0.0001*	0.532 < 0.0001*	0.453 0.0001*	0.256 0.0383*	0.395 0.0010*	0.610 < 0.0001*
mod SAMIS total score				0.377 0.0018*	− 0.309 0.0116*	0.307 0.0123*	0.290 0.0184*	0.146 0.2432*	0.117 0.3487*	0.053 0.6709*	0.290 0.0183*
US-CLARA					− 0.775 < 0.0001*	0.671 < 0.0001*	0.750 < 0.0001*	0.566 < 0.0001*	0.479 < 0.0001*	0.406 0.0007*	0.804 < 0.0001*
HGs peak grip force (kg)						− 0.691 < 0.0001*	− 0.717 < 0.0001*	− 0.586 < 0.0001*	− 0.397 0.0010*	− 0.413 0.0006*	− 0.872 < 0.0001*
CDAI							0.885 < 0.0001*	0.867 < 0.0001*	0.391 0.0012*	0.407 0.0007*	0.713 < 0.0001*
DAS28-CRP								0.838 < 0.0001*	0.403 0.0008*	0.486 < 0.0001*	0.706 < 0.0001*
SDAI									0.311 0.0111*	0.368 0.0023*	0.616 < 0.0001*
AIMS2-HFF										0.211 0.0889*	0.428 0.0003*
Quick-DASH											0.389 0.0012*

*mod SAMIS* modified Simplified Rheumatoid Arthritis Magnetic Resonance Imaging Score; *BME* bone marrow edema; *ERO* erosion; *US-CLARA* UltraSound-CLinical ARthritis Activity index; *HGs* handgrip strength; *CDAI* Clinical Disease Activity Index; *DAS28-CRP* Disease Activity Score 28 joints C-reactive protein; *SDAI* Simplified Disease Activity Index; *AIMS2-HFF* Arthritis Impact Measurement Scale Hand and Finger Function; *QuickDASH* shortened Disability of Arm, Shoulder and Hand questionnaire; *ROAD* Recent-Onset Arthritis Disability questionnaire. Legend: *=p values.

Interobserver agreement was good to excellent for each of the three characteristics (ICC = 0.71, 0.91, and 0.82, respectively, for SAMIS synovitis, SAMIS-BME, and SAMIS-ERO).

## Discussion

In this study, correlations between objective measures of joint inflammation versus HGs and patient-reported measures of function have been demonstrated.

Hand function is generally impaired in the majority of patients with RA. Nevertheless, current recommendations for monitoring disease activity are limited to joint counts, without the inclusion of objective assessment of hand function. Pain and functional limitation may persist despite optimal monitoring of signs of joint inflammation and disease activity [55]. On the other hand,  patient-reported measures are increasingly seen as potentially more reliable than physician-reported measures or laboratory parameters in predicting long-term disease outcomes [56–59]. In this perspective, PROs dedicated to the hand and upper limb have proven to be valid and accurate in assessing the disability of patients with RA [33–35, 45, 60]. There is also evidence that in patients with RA in DAS28 remission or with low compromise of ADLs according to the health assessment questionnaire, HGs is considerably impaired in some cases. HGs is therefore not fully included in traditional indices of disease activity. HGs has been demonstrated to correlate significantly with measures of hand function in patients with RA [61], including the DASH and the AIMS [62, 63]. Reduced HGs has been recognized as a strong predictor of multi-morbidity, disability, and mortality, and is a major contributor to frailty and sarcopenia in young old (aged ≥ 65) and very old adults (aged ≥ 85) [26, 64–67].

Studies have shown both MRI and US to be highly sensitive in assessing the inflammation of joints [68]. However, US is unable to represent BME, a robust predictor of bone damage and disease progression. It may also fail to adequately evaluate certain joint portions [69], and is operator dependent. US has the advantage of being cheaper and more readily available compared to MRI. MRI has the benefit of greater articular coverage and BME detection, but is more expensive and less accessible in a resource-constrained setting [20, 51, 70, 71]. Among patients with active early RA, high levels of objective MRI–detected inflammation at baseline are indicative of which patients are more likely to achieve clinical remission with treatment [72]. For a standardized and easily applicable MRI assessment system, it has developed a scoring system, the EULAR-OMERACT RAMRIS, which includes semiquantitative scores for bone erosion, BME, and synovitis of the wrist and MCP joints [73]. RAMRIS has been simplified in SAMIS in order to overcome the time-consuming aspects and the long learning curve [21, 74]. The modified SAMIS employed for this study graded MRI for the presence/absence of synovitis (SAMIS synovitis) and semiquantitative ratings of bone erosion (SAMIS-ERO) and bone marrow edema (SAMIS-BME), without contrast injection. To save more time, we chose to only assess the one most painful or the dominant hand. MRI, regardless of whether it covers unilateral wrist and MCP joints or bilateral wrists and MCP joints plus unilateral metatarsophalangeal joints, is significantly superior to conventional radiography for the detection of progressive joint destruction in RA [75]. Sufficient reproducibility is a prerequisite for any scoring method: the proposed mod SAMIS had excellent inter-reader reliability.

Several studies have investigated the extent to which RA joint pathologies could be reliably assessed with unenhanced MRI images rather than with gadolinium (Gd)-enhanced MRI (as the reference method) to reduce the imaging time, invasiveness, and cost. Gd is generally recommended for MRI assessment of RA joint changes, particularly synovitis. Previous studies found that the Gd contrast administration  for the MRI procedure did not change the scores of the bone erosion and bone edema but decreased the reliability of the synovitis scores [76–78]. Based on these observations, in this study it has been merely looked at the presence or absence of synovitis without rating it. This could be considered a limitation of the present study, however the use of Gd markedly prolongs the examination time and increases costs, invasiveness, and patient discomfort, and thereby reduces the feasibility of MRI in RA.

There are limitations to mention regarding this study. First, the statistical power was limited by the small sample size of 66 RA patients. Second, there is theoretical concern that generalizability of the mod SAMIS score may be hampered by difficulties in scoring the foot. However, for each of the three features (synovitis, BME, and erosion), interobserver agreement was good to excellent, and this suggests that this generalizability can be applied to other joint regions as well.

In conclusion, BME and synovitis have an influence on the function of the upper extremities. The US-CLARA and the mod SAMIS total score are intriguing options for semiquantitative assessment of joint inflammation and damage in RA, and they are currently being investigated. These shorter scores may reduce the amount of time required for image processing in US and MRI-controlled RA investigations, as well as make the use of these imaging modalities in RA therapy response assessment studies more straightforward. Further longitudinal studies, with larger numbers of patients and using various MRI and US scoring systems, are needed to prove that these methods are universally useful.
